# High quality genome assemblies of *Mycoplasma bovis* using a taxon-specific Bonito basecaller for MinION and Flongle long-read nanopore sequencing

**DOI:** 10.1186/s12859-020-03856-0

**Published:** 2020-11-11

**Authors:** Nick Vereecke, Jade Bokma, Freddy Haesebrouck, Hans Nauwynck, Filip Boyen, Bart Pardon, Sebastiaan Theuns

**Affiliations:** 1 Faculty of Veterinary Medicine, Department of Virology, Parasitology and Immunology, Ghent University, Salisburylaan 133, 9820 Merelbeke, Belgium; 2 Faculty of Veterinary Medicine, Department of Large Animal Internal Medicine, Ghent University, Salisburylaan 133, 9820 Merelbeke, Belgium; 3 Faculty of Veterinary Medicine, Department of Pathology, Bacteriology and Avian Diseases, Ghent University, Salisburylaan 133, 9820 Merelbeke, Belgium; 4 PathoSense, Merelbeke, Belgium

**Keywords:** Basecalling, *Mycoplasma bovis*, Nanopore sequencing, Long-read sequencing, Genome assembly

## Abstract

**Background:**

Implementation of Third-Generation Sequencing approaches for Whole Genome Sequencing (WGS) all-in-one diagnostics in human and veterinary medicine, requires the rapid and accurate generation of consensus genomes. Over the last years, Oxford Nanopore Technologies (ONT) released various new devices (e.g. the Flongle R9.4.1 flow cell) and bioinformatics tools (e.g. the in 2019-released Bonito basecaller), allowing cheap and user-friendly cost-efficient introduction in various NGS workflows. While single read, overall consensus accuracies, and completeness of genome sequences has been improved dramatically, further improvements are required when working with non-frequently sequenced organisms like *Mycoplasma bovis*. As an important primary respiratory pathogen in cattle, rapid *M. bovis* diagnostics is crucial to allow timely and targeted disease control and prevention. Current complete diagnostics (including identification, strain typing, and antimicrobial resistance (AMR) detection) require combined culture-based and molecular approaches, of which the first can take 1–2 weeks. At present, cheap and quick long read all-in-one WGS approaches can only be implemented if increased accuracies and genome completeness can be obtained.

**Results:**

Here, a taxon-specific custom-trained Bonito v.0.1.3 basecalling model (custom-*pg45*) was implemented in various WGS assembly bioinformatics pipelines. Using MinION sequencing data, we showed improved consensus accuracies up to Q45.2 and Q46.7 for reference-based and Canu de novo assembled *M. bovis* genomes, respectively. Furthermore, the custom-*pg45* model resulted in mean consensus accuracies of Q45.0 and genome completeness of 94.6% for nine *M. bovis* field strains. Improvements were also observed for the single-use Flongle sequencer (mean Q36.0 accuracies and 80.3% genome completeness).

**Conclusions:**

These results implicate that taxon-specific basecalling of MinION and single-use Flongle Nanopore long reads are of great value to be implemented in rapid all-in-one WGS tools as evidenced for *Mycoplasma bovis* as an example.

## Background

Long-read sequencing has shown its use in many fields, including metagenomics [1], complete de novo (hybrid) genome assemblies [2], and antimicrobial resistance (AMR) detection [3]. Different single-molecule long-read sequencing approaches have become available nowadays, requiring a relatively small upfront financial investment as compared to Illumina short-read sequencing instrument costs [4]. The latter is still the predominant Next Generation Sequencing (NGS) technology, as highly accurate reads are generated (0.1% substitutions for MiSeq) in comparison to the long-read Oxford Nanopore Technologies (ONT) approach (5% for R9.4.1 flow cell chemistry; ONT communication March 2020) [4, 5]. Nevertheless, highly repetitive genomes tend to pose problems when applying Illumina sequencing owing to read mapping ambiguities, which are also a result of GC biases generated throughout the library preparation protocol [6]. While Illumina short-read sequencing uses the sequencing-by-synthesis approach, nanopore sequencing allows immediate and real-time detection of native or amplified DNA and RNA strands. Upon the insertion of DNA in the nanoscale pore using a secondary motor protein, blockage of the pore-channel allows to measure differential voltages for each k-mer placed within the pore. This results in raw current signals, known as *squiggle spaces*, which allow interpretation of the k-mer sequence stretch blocking the nanopore at a specific moment. Thus, raw nucleotide current signals are generated which harbor the true native sequence information of the DNA [7]. This is of great value for various approaches that require *squiggle* (re-)alignment including basecalling, adaptive sequencing, methylation calling, and their respective training workflows [8, 9].

The generated *squiggles* have to be converted into corresponding stretches of basecalled k-mers. The latter plays a crucial role in further data processing and will aid to overcome high error rates in both single reads and overall consensus sequences. Through the years, various basecalling tools have been developed and released from ONTs bioinformatics R&D line (e.g. Albacore, Guppy, Scrappie, and Flappie) and by third parties (e.g. Chiron [10]). Further development of Albacore and Scrappie has been discontinued, as both Guppy and Flappie were shown better-performing alternatives, respectively. Guppy is a production basecaller available to ONT customers, while Flappie and Chiron are open source basecalling tools, both using a combined Connectionist Temporal Classification (CTC) and Recurrent Neuronal Network (RNN) algorithm [6, 10, 11]. Since a recent study showed Guppy being the best performing basecaller, we will focus on the comparison of Guppy and the in 2019 released Bonito basecaller (ONT) [11]. The latter shows significantly increased median single read Q-scores of Q14.60, resulting in improved consensus accuracies when the pre-built dna_r9.4.1 model is used on datasets for which the model originally was trained for (including *E. coli*, yeast, and human datasets). In order to assess basecalling quality, Q-scores at both read and consensus sequence level can be calculated. While read accuracy represents the number of errors within a single read, consensus sequence Q-scores are a result of overlapping read assemblies compared to the true reference. Hence the latter is in relation with sequencing depth and is considered of greater value in evaluating final whole genome assemblies. Interestingly, Bonito comes with a training module, allowing the generation of custom models through CTC training of the deep neural network (ONT communication, March 2020). Currently the Guppy production basecaller (v.3.4.5) is implemented by default in the MinKNOW software package (release 19.12) allowing fast and high accuracy basecalling. While latest published data on single read and consensus accuracy of Guppy (v3.4.5, High Accuracy (HAC) Model) show median Q-scores of Q13.0 and Q30.0, respectively, major improvements are still required to convert raw current signals from a variety of input sources (e.g. bacterial, viral, or human DNA) to higher levels of single read and consensus accuracy (ONT communication March 2020). Furthermore, training of existing basecalling models was shown to result in significantly increased accuracies. Nevertheless, improvements are only guaranteed if datasets are used for which the model was trained for [11]. Current pre-trained models usually comprise bacterial species (e.g. species present in the Zymo Research Mock Community [12]), yeast, and human datasets, which are broadly studied in the genomics field, resulting in high accurate overall de novo assembled consensus genomes, reaching scores beyond Q40 (ONT communication, January 2020). Even though a wide variety of known relevant bacterial species are chosen for model training, taxon-specific variations in genome build-up, such as repeat regions, insertion sequences, extreme GC contents, and distinct nucleotide modifications (e.g. methylation patterns) are known to limit basecalling with standard pre-trained models [6, 11]. When applying the default Guppy basecaller to less frequently sequenced bacteria, such as *Mycoplasma* spp., overall consensus accuracy does not reach the aimed qualities. This can be addressed to diverging genome characteristics as compared to bacteria frequently used in genomic analyses.

Here, the *M. bovis* species was chosen as representative for the *Mycoplasma* genus and clinically relevant primary respiratory pathogen in veterinary medicine. *M. bovis* is a bacterium belonging to the *Mollicutes* class, lacking a cell wall and therefore showing natural resistance against beta-lactam antibiotics [13]. Furthermore, *M. bovis* is highly contagious and results in enormous economic losses in the cattle sector. Since no vaccination strategy has been shown effective up to date, antimicrobials are frequently used to control *M. bovis* infections. This results in an increased risk of resistance to other commonly used antimicrobials in both *M. bovis* and other cattle-associated bacteria [13, 14]. On the genetic level, the *M. bovis* genome exhibits peculiar characteristics with a GC content of barely 29.4%, presence of many highly repetitive regions, and distinct *6 mA* methylation sites [15, 16]. Furthermore, high adenine–thymine pressure results in the use of translation table 4, rather than the standard bacterial translation table 11 [16]. A variety of molecular- and culture-based *M. bovis* identification tests have been developed to improve speed, sensitivity, and specificity. However, none of them allow quick and simultaneous identification, strain typing, and AMR profiling [17, 18]. Current diagnostics require at least 6 days before a pure *M. bovis* culture can be obtained. Thus, rapid and complete diagnostics tools are required [18]. Previously, MinION sequencing has been shown valid in phylogenetic analyses and AMR prediction of *Neisseria gonorrhoeae* and *Mannheimia haemolytica* in human and veterinary diagnostics, respectively [3, 19]. The development of rapid and cheap revolutionizing diagnostics tools requires extensive validation and potential implementation of cost-efficient state-of-the-art sequencing devices, including single-use Flongle R9.4.1 chemistry flow cells, and innovative bioinformatics tools. The use of the cost-effective disposable Flongle flow cell was previously demonstrated for metagenomics and AMR analyses in a preterm point-of-care hospital setting [1].

The goal of this work was to verify the use of a *M. bovis* PG45 type strain custom-trained Bonito basecalling model (custom-*pg45*) within a complete bioinformatics workflow to increase final consensus sequencing accuracies. This will allow further implementation of a rapid and cost-efficient full *M. bovis* identification, strain typing, and AMR detection diagnostics workflow in the veterinary field. To this end, a comparative analysis of MiSeq Illumina short reads versus ONT long reads from both MinION and single-use Flongle R9.4.1 flow cells was performed, using the state-of-the-art basecalling, mapping, and genome assembly tools, available at the time of manuscript submission.

## Results

### Improved reference-based and de novo assemblies using an M. bovis trained Bonito basecalling algorithm

**Fig. 1:**
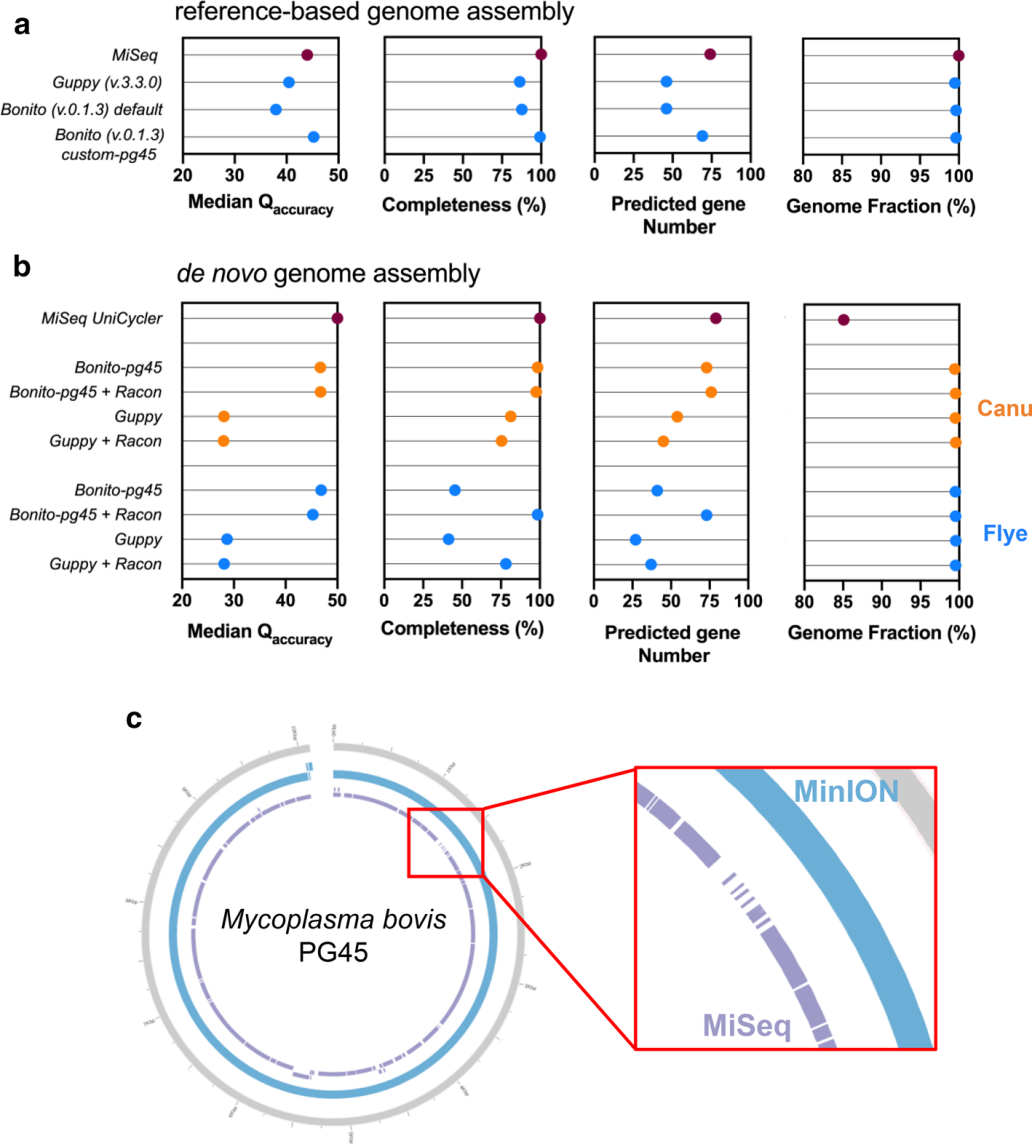
Implementation of custom-*pg45* Bonito training in reference-based (**a**) and de novo genome assembly bioinformatic workflows (**b**). The newly generated custom-*pg45* Bonito training model was implemented in both reference-based (**a**) and de novo assembly (**b**) bioinformatics workflow. While for MiSeq short reads (purple), UniCycler (SPAdes-based) de novo assembler was used, long reads were used in a bioinformatics pipeline with either the Canu (orange) or Flye (blue) de novo assembler, supplemented with or without four rounds of Racon polishing. **c** MiSeq sequencing results in a highly gapped de novo assembled *M. bovis* PG45 genome as compared to MinION long read assemblies. A completeness of 100% indicates all genomic markers (n = 226 for *Mycoplasma* spp.) were present. A 100% Genome Fraction indicates the full *M. bovis* PG45 type strain genome was covered

Performing basecalling with a custom-trained Bonito v.0.1.3 model (custom-*pg45*), using taxon-specific training data (from unamplified DNA of the *M. bovis* PG45 type strain sequenced on MinION) resulted in a significantly increased final consensus accuracy for reference-based genomes (Q45.2) (Fig. 1a). When comparing with the MiSeq approach, a + Q1.3 was shown for the Bonito-generated data, along with improved genome completeness and predicted gene content, reaching to 99.3% and 69 out of 75 precited genes. The latter confirms plausible genome assemblies were obtained, since similar numbers of biologically relevant genes were found as compared to the 75 predicted genes within the *M. bovis* PG45 reference. As expected for a reference-based approach, all resulting *M. bovis* PG45 genomes covered the reference for at least 99.5%. (Fig. 1a).

As shown in Fig. 1b, similar improvements were seen for de novo assembled genomes. Still, a -Q3.3 was shown compared to the MiSeq UniCycler de novo assembly. Assemblies generated by two widely used long read de novo assemblers, Canu and Flye, were compared, eventually supplemented with four rounds of Racon-GPU polishing. Both Canu (Q46.7) and Flye (Q46.9) performed evenly good in generating high accuracy consensus genomes when custom-*pg45* basecalling was implemented without racon polishing. Nevertheless, a clear lack of genome completeness (45.5%) and predicted genes (41 out of 79 genes) was observed for the Flye assembly as compared to a 98.5% genome completeness and 73 out of 75 predicted genes using Canu. Improved completeness (8.0% to 82.6%) and predicted gene numbers (41 to 73 out of 79) were obtained when Flye assemblies were submitted to four rounds of Racon-GPU polishing (Fig. 1b).

Even though the MiSeq short read UniCycler de novo assembly performed better in both consensus accuracy (Q50.0) and genome completeness (100.0%), only 85.1% of the *M. bovis* PG45 reference was covered as compared to assemblies generated by long reads (on average 99.5%). The latter showed similar genome fractions as observed for reference-based approaches (Fig. 1b). When looking at resulting contigs, a multitude of MiSeq contigs resulted in highly gapped *M. bovis* PG45 genome, whereas long read MinION contigs fully covered the reference genome (Fig. 1c). Further comparative analyses were performed using the Canu assembler without four rounds of Racon instead of the Flye with four rounds of Racon approach. Even though similar performance was observed for both bioinformatics pipelines, computational speed and occurrence of less mismatches and indels are in favor of the Canu approach (Additional file 1: Figure S1).

## Validation of the M. bovis trained basecalling model using *E. coli* and *M. bovis* field strains

**Fig. 2:**
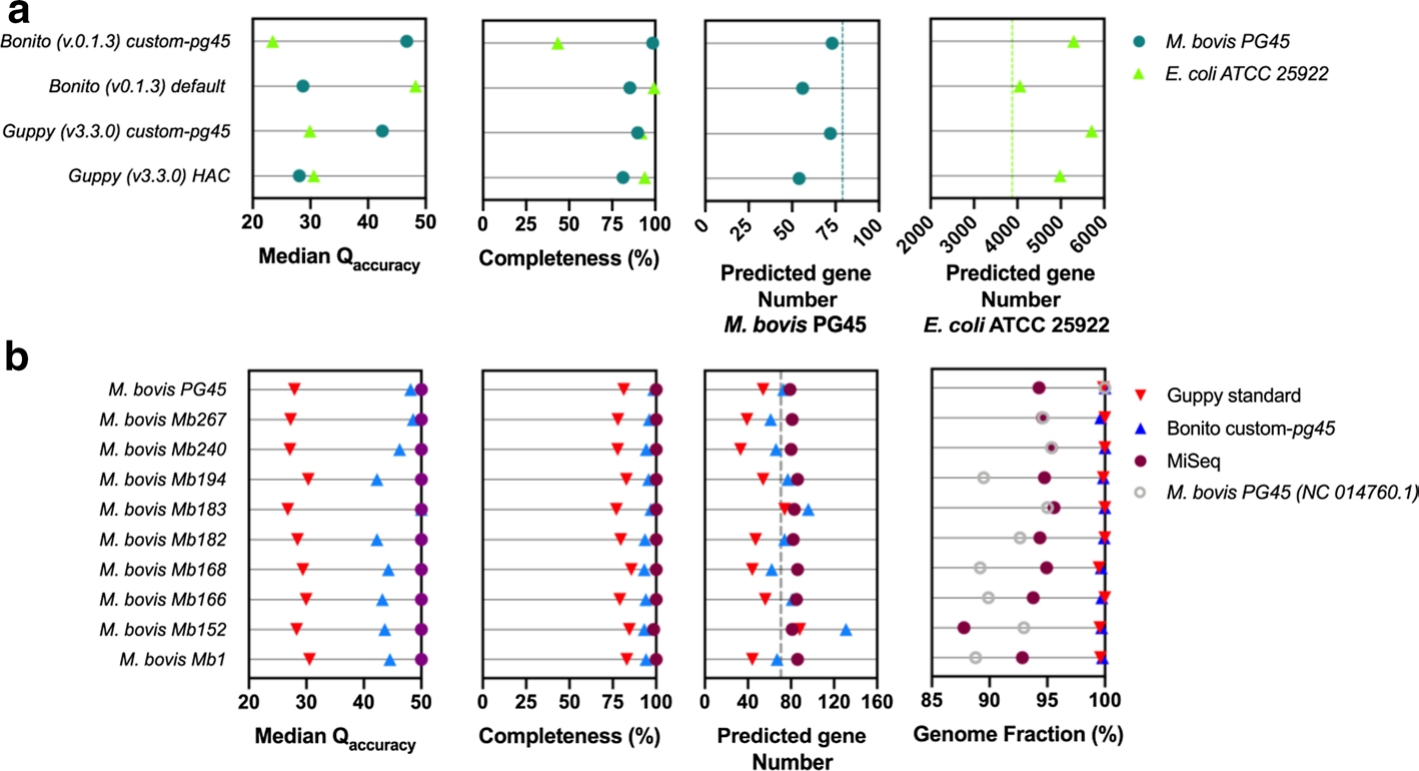
Validation of the specificity of taxon-specific custom-*pg45* Bonito basecalling using **a** *E. coli* ATCC 25922 and **b** nine additional *M. bovis* field strains. **a** Performance of the *M. bovis* PG45 custom-trained Bonito model was tested in comparison with *E. coli* ATCC 25922, only showing taxon-specific superior performance. Dotted lines represent predicted gene numbers in *M. bovis* PG45 and *E. coli* ATCC 25922 reference genomes, respectively. **b** Extrapolation of the custom-*pg45* implementation to nine additional *M. bovis* field strains, shows overall increased performance for all strains in comparison to default Guppy basecalling. As a matter of validation of the UniCycler consensus genomes as “true” references, the *M. bovis* PG45 (NC_014760.1) was included in the analysis and indicated in grey. A completeness of 100% indicates all genomic markers (n = 226 and n = 1212 for *Mycoplasma* spp. and *Escherichia* spp., respectively) were present. A 100% Genome Fraction indicates the full UniCycler MiSeq *M. bovis* field strain genome was covered

In order to validate the specificity and implementation of the taxon-specific custom-*pg45* Bonito basecalling approach in the Canu-based bioinformatics workflow, de novo consensus genomes for both *M. bovis* PG45 and *E. coli* ATCC 25922 were generated using either Guppy, default Bonito dna_r9.4.1 basecalling model, or Guppy and Bonito custom-*pg45* basecalling model. Overall consensus accuracy of the default Guppy basecalled genomes did not result in major median Q-score differences (Q28.1 and Q30.6 for *M. bovis* and *E. coli*, respectively). Furthermore, none of the resulting genomes reached above 86% genome completeness. Implementing the default dna_r9.4.1 Bonito model for *M. bovis* PG45 de novo genome assemblies did only result in a slight improvement of genome completeness (81.3% to 85.4%). Nevertheless, highly improved consensus *E. coli* ATCC 25922 genomes were generated reaching to Q48.2 consensus accuracy, 99.3% genome completeness, and 4057 out of 3877 predicted genes. Presence of more predicted genes can be explained by duplicated regions within the obtained *E. coli* ATCC 25922 long read consensus genome. Inversely, when using the custom-*pg45* basecalled reads, the *M. bovis* PG45 consensus sequence reached up to Q46.7, 98.5% genome completeness, and 73 out of 79 predicted genes. Applying the custom-*pg45* model on *E. coli* ATCC 25922 led to worse results compared to the Guppy basecalled consensus genome. Custom training for Guppy basecalling did also result in increased quality of *M. bovis* genomes (Q42.5 and 89.8% complete). However, custom-*pg45* Bonito basecalling still outperformed Guppy (Fig. 2a).

Further validation of the custom-*pg45* Bonito model was performed on nine additional *M. bovis* field strains to see its valid implementation in veterinary diagnostics. Here, UniCycler MiSeq genomes were used as reference sequences to determine Q-scores, genome fractions, and predicted genes. To verify this, the *M. bovis* PG45 (NC_014760.1) reference strain genome was also analyzed along with MiSeq and MinION consensus genomes. MiSeq short read UniCycler assemblies resulted in overall consensus accuracies of Q50, at least 98.0% genome completeness, and on average 83 (± 2 (SD)) predicted genes for all field strains. Again, superior performance was observed when custom-*pg45* Bonito basecalling was implemented, which was in contrast to standard Guppy basecalling results on these ten *M. bovis* strains (Fig. 2b). These data are in line with our previous *M. bovis* PG45 analysis (Fig. 1b). When compared to MiSeq accuracies, a range of less accurate -Q7.7 up to equally accurate (-Q0) Q-scores were observed for different *M. bovis* field isolates. Furthermore, all field strain genomes, generated with taxon-specific basecalling, resulted in genome completeness scores ranging between 93.2% and 98.5% as compared to a range of 77.0% up to maximal 85.3% when Guppy basecalling was used. These trends are also observed in the predicted gene numbers (79 ± 22 of 83 ± 2 for different MiSeq assemblies). Again, presence of more predicted genes can be explained by duplicated regions within the final *M. bovis* consensus sequence. Additionally, different *M. bovis* field strains potentially harbor various numbers of core genes as compared to the used *M. bovis* PG45 type strain since an order-level database is used (mycoplasmatales_odb10 (n = 174)). Again, genome fractions of MiSeq data showed overall lowered coverage when short reads were used instead of nanopore long reads. Furthermore, including the ncbi reference strain granted supplementary information on genome size diversity among analyzed *M. bovis* field strains, ranging from 88.8% up to 95.4% for the Mb1 and Mb240 isolates, respectively (Fig. 2b).

## Application of disposable single-use Flongle flow cells in an optimized *M. bovis* bioinformatics workflow for veterinary diagnostics

**Fig. 3:**
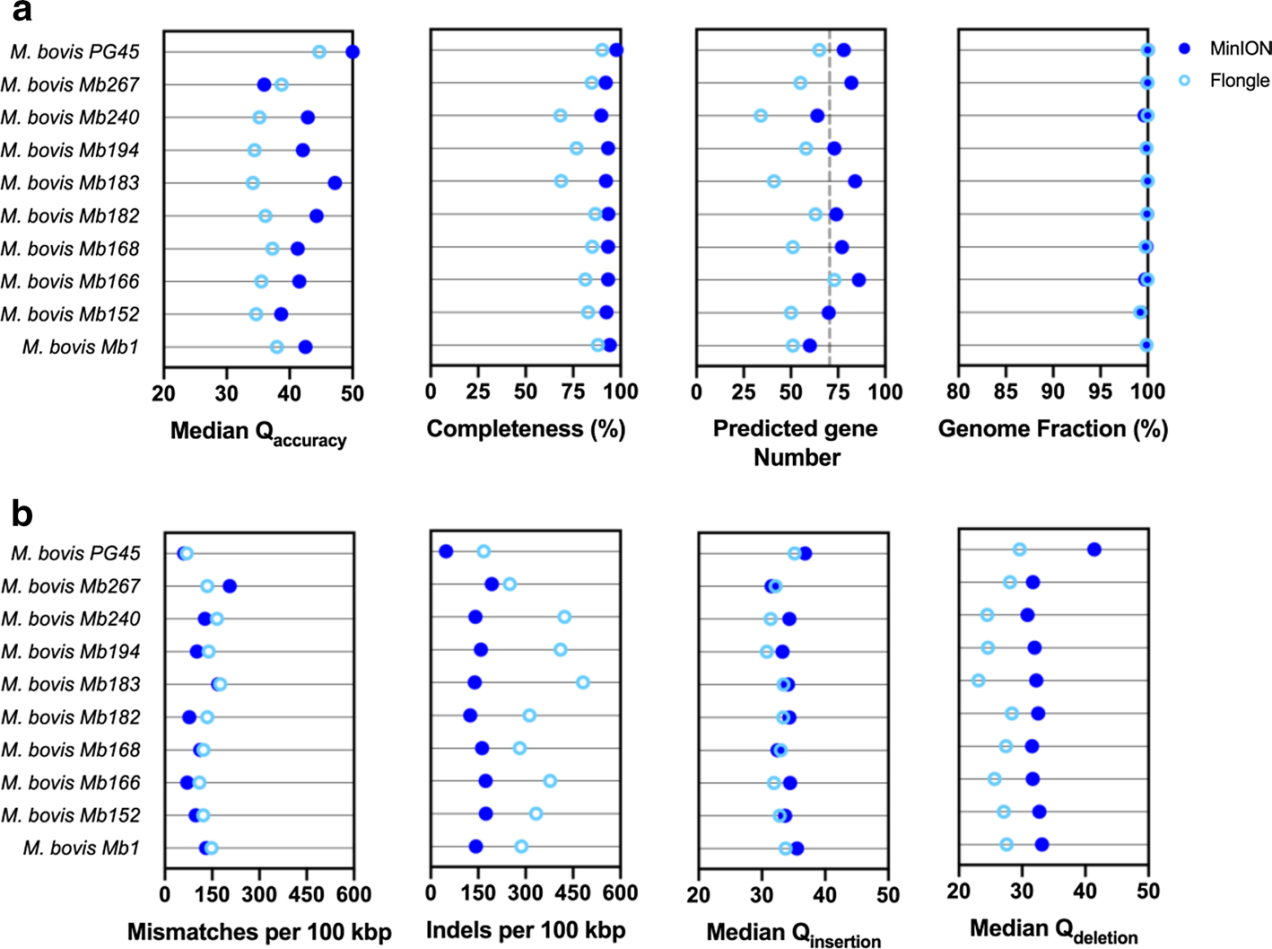
Comparative analysis for the implementation of cheaper single-use Flongle flow cells, using the custom-*pg45* model. **a** Overall Flongle consensus accuracy Q-scores are lower as compared to the MinION sequencing platform. This is also reflected in lowered genome completeness and predicted gene numbers. **b** In-depth analyses of decreased Flongle performance is suggested to be related to increased numbers of indels (per 100 kbp), showing little differences in insertion Q-score, though a decrease of accuracy for reported deletions is observed. A completeness of 100% indicates all genomic markers (n = 226 for *Mycoplasma* spp.) were present. A 100% Genome Fraction indicates the full UniCycler MiSeq *M. bovis* field strain genome was covered

In respect to potential integration of long-read sequencing platforms in veterinary diagnostics, rapid, cheap, and accurate workflows are at stake. Here, we tested the potential of a cheaper disposable single-use Flongle flow cell (with same R9.4.1 Nanopore chemistry) within the optimized custom-*pg45* Bonito basecalling Canu-based bioinformatics workflow. To allow proper comparison, MinION-sequenced reads of the *M. bovis* PG45 type strain and nine field strains were subsampled to obtain comparable coverage as for Flongle sequencing runs (Additional file 2: Table S3). Best results were obtained for *M. bovis* PG45 on both MinION (Q50.0) and Flongle (Q44.7) sequencing platforms. This is a result of training the model with *M. bovis* PG45 data only, as only for the type strain a reference sequence was available. Overall consensus Q-scores of the nine *M. bovis* field strains were better when using MinION reads. Q-score differences seemed to vary depending on the strain with mean Q-scores of Q36.0 ± 1.6 as compared to the better performing MinION output (Q41.8 ± 3.2). Similar results were obtained when looking at genome completeness (80.3 ± 7.4%) and predicted genes (53 ± 11 out of 74 ± 9 predicted genes) (Fig. 3a). In the case of Mb267, better Flongle assemblies were generated, which is suggested to be due to the low coverage (< 50X) and potential bias generated through the applied subsampling method. Noteworthy, subsampled MinION reads resulted in non-significantly lower (*p* = 0.0704) consensus Q-scores as compared to when all MinION reads were used in the analysis (Q45.0 ± 2.7) (Figs. 2b, 3a). Even though slightly lower consensus Q-scores were obtained, use of disposable single-use Flongle flow cells in point-of-care bacterial diagnostics settings is of major interest for *M. bovis* identification and association of AMR in the veterinary field. While identification of AMR-related genes and/or point mutations is facilitated by whole-genome sequencing diagnostics, implementation of this custom-trained model contributes to increased resolution to draw comprehensive conclusions. In order to fully implement this approach, functional studies linking genotypes and phenotypes should be further addressed in depth.

In order to better understand the potential origin of the slightly lower performance using Flongle, further in-depth Median Q-scores were calculated, pointing to lowered overall consensus Q-scores rather due to significantly more indels (187 ± 86 per 100 kbp more in Flongle), but not mismatches (on average 31 ± 22 per 100 kbp more in Flongle). When looking into insertion and deletion Q-scores, deletion accuracies (Q26.6 ± 2.1) were significantly lower as compared to the use of MinION reads (Q33.0 ± 3.0) (Fig. 3b).

## Discussion

With the release of the Convolutional Neural Network based Bonito basecaller (ONT) in December 2019, significant improvements of consensus accuracies were observed as compared to the default Guppy High Accuracy basecalling algorithm (ONT communication, March 2020). Interestingly, the Bonito training module allows CTC-driven training of the given model. With approximately only 20 and 131 median errors in a 1 Mbp genome for reference-based and de novo assembled *M. bovis* PG45 genomes, respectively, we are getting very close to “perfect” consensus genomes using the Nanopore sequencing technology. Here, the use of taxon-specific training of the Bonito basecalling model was shown of major value when basecalling raw *squiggle spaces* originating from both MinION and Flongle flow cells when working with *Mycoplasma bovis*. Still further improvements have to be made to get to more accurate genomes, which can be obtained by improving the quality of deletions and insertions across the genome. To do so, proper choice of available state-of-the-art bioinformatics tools within different pipelines is of major importance as well as exploiting taxon-specific training of the available tools. New releases of consensus polishing tools, such as Medaka v.0.12.1 (ONT) do also allow taxon-specific training ( [2] and medaka benchmark ONT 2019). Further single read and consensus accuracies have been improved by the release of the new Guppy v.3.6 (with a 1–2% increase in single read accuracy) (ONT communication May 2020) and the new R10.3 chemistry flow cells, reaching accuracies above 99.999% as compared to the 99.99% for the current R9.4.1 flow cells [32]. One might also want to implement novel algorithms to align raw *squiggles* or use simulations of reads generated by DeepSimulator or NanoSim in order to evaluate newly generated models or to even further reduce specific base calling errors [33–35].

Long-read sequencing by Oxford Nanopore Technologies provides quick, cheap, innovative, and compact sequencing solutions which have major potential to be implemented in various WGS applications, including metagenomics, AMR detection, and viral/bacterial diagnostics. However, major culprit for extended implementation lies within single read and consensus accuracy. Currently, Illumina (e.g. MiSeq) short-read approaches are still predominating the NGS field, although genome assemblies using short reads were shown to result in ambiguous read mapping when the genome of interest exhibits features such as highly repetitive regions, insertion sequences, extreme GC contents, and/or distinct methylation patterns, as frequently observed in *Mycoplasma* spp.[6, 16, 36]. While hybrid-assembly approaches, combining both long and short read data, showed improved genome assemblies [2], a hybrid-assembly approach is not preferred in point-of-care human and veterinary diagnostics, since rapid and complete diagnostic tests should be provided at a low cost, but high accuracy. Thus, improving bioinformatics pipelines by implementation of custom-trained basecalling models has previously shown very promising to circumvent the need of short read sequencing in Hybrid assemblies [11]. Here we showed significantly increased overall consensus accuracies and more complete genomes for both reference-based and de novo assembled *M. bovis* genomes when using the custom-*pg45* Bonito v.0.1.3 model. Furthermore, implementation of the Bonito default basecaller resulted in a higher quality *E. coli* genome (99.1% genome completeness) using significantly lower read input (1.65 Gbps) and a simple bioinformatics workflow, as compared to results from Nicholls et al*.* (2019). They reached a genomic completeness of 97.1% using PromethION reads (11.62 Gbps) and an extended bioinformatics pipeline (2 round of Racon, Medaka, and 2 rounds of Pilon polishing) [12]. Nanopore sequencing allows analysis of native unamplified DNA, resulting in raw read current signals comprising metadata beyond nucleotide sequences. Those signals additionally provide information of modifications, including epigenetic methylation patterns (e.g. *Dam* and *Dcm* methylations) [37]. This has previously been proven crucial for correct long read basecalling, as stretches of k-mer nucleotides and not single bases are analyzed within the nanopore. Previously, custom-training of the default Guppy v2.2.3 model has shown to increase single read and consensus accuracy in *Klebsiella pneumoniae*, a commonly studied member of the Enterobacteriaceae [11]. While bacterial epigenetics have only been discovered recently, it is thought to play crucial roles in virulence, AMR, DNA regulation, and defense mechanisms. Interestingly, *6 mA* methylation is widespread within the kingdom of bacteria and many *6 mA* methyltransferase families have been found across diverse bacteria, each resulting in methylation of different DNA patterns [38]. Thus, taxon-specific methylation sites are suggested to be at the base for improved basecalling performance with taxon-specific trained models. The same accounts for the *Mycoplasma* sp. model, where *6 mA* methylations have been shown at 5′-CTAT-3′ sites, which differ from the Gammaproteobacteria-specific 5′-GATC-3′ site. Additionally, more complex methylation sites have been described for *Mycoplasma* sp.[36]. With raw long-read sequences originating from native *M. bovis* DNA, these data can contribute to understand the importance of *M. bovis* specific methylations. Megalodon (v2.0 released in March 2020 by ONT), a bioinformatics tool allowing the extraction of high accuracy modified bases, might be of specific interest for such application. However, to date Megalodon is only compatible with flip-flop basecalling networks (e.g. Guppy) and cannot be used with Bonito yet. One of the suggested crucial relationships between taxon-specific methylation patterns and basecalling accuracy is further supported by the fact that improved consensus accuracies were observed for nine non-type strain *M. bovis* field strains and not for the *E. coli* ATCC 25922 strain, when a custom-*pg45* basecalling model was implemented. Since bacterial methylation events have been linked to raising incidence of AMR, implementation of native long-read DNA sequencing approaches in human and veterinary bacterial identification, typing, and AMR detection might result in revolutionizing all-in-one high-resolution diagnostics tools in both human and veterinary medicine.

Current molecular- and culture-based *M. bovis* diagnostic tools are time-consuming and provide only partial information, obscuring prompt disease control and prevention [17]. Current default *M. bovis* identification diagnostics, imply the use of single or multiplex (q)PCR approaches, showing up to 100% specificity. Nevertheless, cross-reactivity has been observed with co-existing non-pathogenic *Mycoplasma* sp. and the sample processing workflow does not allow further in-depth analyses [39]. Recently, a cheaper, though less specific (86.4%), MALDI-TOF MS culture-based method was investigated and shown promising for routine diagnostics testing, allowing further AMR detection and genotyping from the same sample [18]. Nevertheless, none of the currently available diagnostic tools allows quick and accurate combined *M. bovis* identification, genotyping, and AMR detection. This might be addressed using WGS approaches, as already described in both veterinary and human clinical settings, including *M. haemolytica* and *N. gonorrhoeae*, classified as AMR “priority pathogens” by WHO [3, 19]. As such, we investigated the potential implementation of a sequencing-based workflow using MinION R9.4.1 flow cells and the single-use Flongle R9.4.1. Overall, lower consensus accuracies were obtained for Flongle-sequenced reads which were basecalled using the Bonito custom-*pg45* model. Strain-dependent overall consensus Q-scores were observed between *M. bovis* field strains, which is possibly a result of high plasticity of the *M. bovis* genome, resulting in differences such as more diverse repetitive regions and potential new/altered methylation sites [36, 38, 40]. Noteworthy, the observed lowered performance might be explained by the use of the *M. bovis* PG45 type strain sequence (generated with the Sanger random shotgun method), only showing an 8X coverage [41]. Using this sequence as “true” reference might result in a potential bias. It is suggested to further investigate whether a more accurate *M. bovis* PG45 genome could become available as an updated standard for genomics studies in *M. bovis*. Even though both flow cell types exploit the R9.4.1 nanopore chemistry, current raw data might not be converted into similar *squiggle spaces* due to differences in underlying bioinformatics code within the MinKNOW software package. Our generated data suggest differences in deletions rather than insertions and mismatches. The provided new insights might aid in further research to lift Flongle sequencing output to MinION scale. In our hands, the stability of the Flongle nanopores still resulted in variable sequencing output for *M. bovis* (57.5—321.6 Mbp in 24 h), which has to be taken into account when implemented in experimental workflows. Elevated Flongle sequencing depth might further improve sequencing accuracies, as slightly better consensus accuracy Q-scores were observed with all MinION reads as compared to the subsampled MinION fraction. While the Flongle platform is suggested to already allow sufficient resolution for *M. bovis* identification, typing, and phylogenetic analyses, its implementation in accurate AMR point mutation scale detection still has to be further determined. Further improvements on the level of both flow cell chemistry and bioinformatics tools might be required here. Nevertheless, the presented data, including the new R9.4.1 chemistry and custom-*pg45* model has shown to be both rapid and accurate and opens the door to its use in all-in-one *M. bovis* diagnostics tools on the MinION platform.

## Conclusions

In this work we have presented a usefull means of obtaining high quality genome assemblies from long-read nanopore sequencing data. In particular, we showed improved *Mycoplasma bovis* genomes by implementing a species-specific trained Bonito basecaller model in a complete bioinformatics workflow.

The way how we approached the issue of lowered accuracy of nanopore long-read sequencing allows to generate both complete and high quality genomes from long-read sequencing data only. Hence, our method shows that hybrid approaches (i.e. combining short- and long-read sequencing data) are no longer necessary to improve genome completeness and quality when using nanopore sequencing. Implementation of multiple field strains supported the robust quality improvement of genome assemblies on both MinION and Flongle sequencing platforms, highlighting its further implementation in veterinary diagnostics for bacterial identification, typing, and potential AMR genotyping. Finally, our method is not limited to *Mycoplasma bovis* and can as such easily be extrapolated to any bacterial species of interest.

## Methods

### Bacterial growth, DNA isolation, library preparation, and sequencing workflows

Nine *M. bovis* field strains and the *M. bovis* PG45 Type Strain (ATCC 25523) were grown from culture using basic PPLO broth (BD Diagnostic Systems), supplemented with 0.5% sodium pyruvate (Sigma-Aldrich), 0.5% D-(+)-glucose monohydrate (Sigma-Aldrich) and 0.005% phenol red [20]. High-molecular weight (HMW) DNA of these cultures was isolated using the ZymoBIOMICS DNA miniprep kit (Zymo Research) [21]. For comparative studies, the *E. coli* ATCC 25922 was grown on a blood-agar and colonies were picked and re-suspended in 250 μL dPBS (Gibco), followed by DNA isolation. Nanodrop 2000 spectrophotometry was used to determine quantity and quality of resulting DNA. Low quality DNA was further cleaned using CleanNGS beads (CleanNA). Subsequently, DNA was aliquoted for MiSeq, MinION, and Flongle sequencing, respectively. MiSeq sequencing was performed by MacroGen (South-Korea), whereas MinION and Flongle sequencing were performed by PathoSense (Belgium). Illumina libraries were prepared using the Nextera XT library kit, whereas ONT library preparations were done using the Rapid Barcoding Kit (SQK-RBK004, ONT). As input, 400 ng and 200 ng HMW DNA was used for MinION and Flongle libraries, respectively. Ten barcoded strains were multiplexed on MinION (48 h sequencing with max. 512 available sequencing channels), whereas single strains were sequenced on Flongle (24 h sequencing with max. 126 available sequencing channels). In both cases R9.4.1 flow cells were used.

## Training a Bonito basecalling model with custom *M. bovis* PG45 type strain *squiggles*

A custom Bonito model was trained using the MinION raw current signal fast5 file from a multiplexed 48 h MinION sequencing run, including the *M. bovis* PG45 type strain. First, 124,856 M*. bovis* PG45 specific read IDs were extracted from Guppy-basecalled data in order to extract *M. bovis* PG45 reads from the fast5 file using fast5_subset, part of the ont_fast5_api package (v.3.1.1; ONT). Subsequently, Guppy-basecalled reads were aligned to the “true” *M. bovis* PG45 reference (NC_014760.1) genome using guppy_aligner (v.3.3.0; ONT) allowing to link extracted read IDs to its “true” reference sequence in the subsequent Taiyaki training workflow (v.5.0.0; ONT). The Taiyaki bioinformatics pipeline is used to prepare the required data for training with Bonito, including prepare_mappeds_read.py to link raw squiggles to its “ground truth” reference based on the guppy_aligner alignment output and raw fast5 file. A fork of the Taiyaki workflow can be accessed on Github (https://github.com/pathosense/taiyaki). Here the r9.4.1_dna_minion Guppy model was given as input for future custom training with the MinION *M. bovis* PG45 dataset. The resulting files, in chunkify format, were converted to npy files by the Bonito convert.py script. Those files were then used for training the custom Bonito model, further named custom-*pg45*, through Bonito train (v.0.1.3; ONT), using the –amp –batch 512 –chunks 1,000,000 –epochs 75 –multi-gpu options. Custom training for Guppy basecalling was done using the same starting model (r9.4.1_dna_minion), following the complete Taiyaki workflow using the train_flipflop.py module, specifying –chunk_len_min 1000 –sample_nreads_before_filtering 1,000,000. Actual training was done on the Ghent University High Performance Computer Tier 2 GPU cluster Joltik, using a single node with 4 × NVIDIA Volta V100 GPUs (32 GB GPU memory each) to speed up the training process. The resulting trained model was used for Bonito basecalling (v.0.1.3; ONT) of both MinION and Flongle datasets.

## Raw long-read basecalling using Guppy and (custom-*pg45*) Bonito basecallers

Basecalling of MinION and Flongle raw signals was done using Guppy (v.3.3.0; ONT), on a local GPU resource (NVIDIA GeForce RTX 2080 Ti GPU (11 GPU memory)). Bonito basecalling was performed using the default pre-trained dna_r.9.4.1 or custom-*pg45* model on the Ghent University HPC Tier 2 GPU cluster Joltik using 1 × GPU. As current Bonito basecalling did not include quality values at the time of writing, resulting fasta files were reformatted to fastq files giving an artificial Q-score of 12 to each base prior to further use in reference-based or de novo genome assembly pipelines. This does not affect any further analyses but is required due to default fastq input requirements of various bioinformatics workflows.

## Reference-based genome assembly

Reference-based genome assemblies were generated for both MiSeq short reads and MinION long-reads, using the full circular *M. bovis* genomic sequence generated with the Sanger random shotgun method (NC_014760.1; coverage: 8X). Quality filtering and adapter trimming of MiSeq reads was performed using Trimmomatic (v.0.39; [22]), specifying ILLUMINACLIP:NexteraPE-PE.fa:2:30:10 LEADING:3 TRAILING:3 MINLEN:45 options. Paired filtered short reads were subsequently mapped to the reference genome using bowtie2 (v.2.2.5; [23]). MinION long raw reads were demultiplexed, filtered and quality trimmed using qcat (v.1.1.0; ONT) and NanoFilt (v.2.5.0; [24]), respectively. Filtered long reads were subsequently aligned using Graphmap (v.0.5.2; [25]). For both short and long read datasets, final consensus sequences were generated using Medaka (v.0.12.1; ONT). Complete genome contigs (> 1 Mbp and > 5 Mbp for *M. bovis* and *E. coli*, respectively) were extracted. Otherwise all contigs were used for subsequent analyses. Summaries can be found in Additional files 2: Table S3, 3: Table S1, 4: Table S2.

## De novo genome assembly

De novo genome assemblies were performed on MiSeq, MinION, and Flongle reads. Since Unicycler (v.0.4.9b) is seen as a state-of-the-art (hybrid) short-read de novo assembler [26], implementing SPAdes de novo assembly and various rounds of Pilon polishing, it was used to generate de novo *M. bovis* sequences using quality filtered and adapter trimmed MiSeq reads. For both MinION and Flongle long-read sequencing outputs two de novo assemblers were compared: (1) Canu (v.1.9; [27]) and (2) Flye (v.2.6; [28]). Since consensus sequence polishing showed possible improved de novo assembly accuracy, four rounds of Racon-GPU polishing (v.1.4.9; [29]) were tested. Final consensus sequences were generated using Medaka (v.0.12.1; ONT). Comparative analyses using *E. coli* ATCC 25922 MinION data and datasets from nine additional *M. bovis* strains (MinION and Flongle) were performed using Canu assembler without Racon as this was shown to result in most complete and highest accurate de novo genome assemblies. In order to properly compare MinION and Flongle assemblies, MinION reads were subsampled to correspond to Flongle read numbers (Additional file 2: Table S3).

## Overall consensus accuracy metrics calculation and genome completeness analysis

Consensus accuracy metrics were calculated using the assess_assembly component of the Pomoxis environment (v.0.3.2; ONT), by aligning 100 kb chunks to the *M. bovis* PG45 (NC_014760.1) or *E. coli* ATCC 25922 (NZ_CP009072.1) reference genomes. Since no “true” reference genomes were available for the nine *M. bovis* field strains, UniCycler de novo assembled genomes, using MiSeq short reads to map chunks. This was validated by including the ncbi *M. bovis* PG45 type strain sequence in the analyses. Q-scores are represented as median values over all chunks with a max. Q-score of 50.00 shown. Further in-depth analysis was performed using QUAST (v.5.0.2; [30]), including indels and mismatches per 100 kbp, genome fractions, and gene predictions (through glimmerHMM). A 100% genome fraction indicates the full reference genome was covered. Supplementary genome quality was assessed using CheckM (v.1.1.0; [31]) by checking for *Mycoplasma* spp. (n = 226 markers from 83 genomes) and *Escherichia* spp. (n = 1212 from 27 genomes) specific marker sets. Here, a 100% completeness indicates all genomic markers were present in the assembly. All data were analyzed and visualized using GraphPad Prism v.8 and values are represented as mean ± Standard Deviation (SD). Statistical significant differences (*p* < 0.05) were determined using a paired t-test.

## Supplementary information

**Additional file 1: Figure S1**. In-depth analyses of de novo consensus genomes between the Canu and Flye assemblers, with or without four rounds of Racon. While both Canu and Flye result in a similar number of mismatches (per 100 kbp), significantly more indels (per 10 kbp) are observed when implementing the Flye assembler in the bioinformatics workflow. Decreased Flye performance is suggested to lie within decreased insertion accuracies as compared to Canu. Implementation of four rounds of Racon allows increased Flye insertion Q-scores when custom-*pg45* basecalling is used. However, Canu without four rounds of Racon still results in an overall better performance.

**Additional file 2: Table S3**. Sequencing summary of single *M. bovis* sequencing runs (24h) on Flongle R9.4.1 flow cells and resulting coverages after qcat and NanoFilt filtering and trimming.

**Additional file 3: Table S1**. Sequencing summary of multiplexed *M. bovis* and *E. coli* sequencing runs (48 h) on MinION R9.4.1 flow cells and resulting coverages after qcat and NanoFilt filtering and trimming..

**Additional file 4: Table S2**. Sequencing summary of the *M. bovis* MiSeq (Illumina) sequencing run with resulting genome coverages after quality-filtering and adapter-clipping.

## Data Availability

Raw filtered sequencing read files have been deposited under ENA Project Accession PRJEB38523 (https://www.ebi.ac.uk/ena/data/view/PRJEB38523), as seven different samples. ERS4590717 (Flongle_Bonito_basecall_All_strains), ERS4590716 (Flongle_Guppy_basecall_All_strains), ERS4590718 (MinION_Bonito_basecall_All_strains_Subsampled), ERS4590715 (MinION_Bonito_basecall_All_strains), ERS4590714 (MinION_Guppy_basecall_All_strains), ERS4590776 (Comparison_M_bovis_E_coli_All_basecallers), ERS4590775 (MiSeq_All_strains). The authors confirm all supporting data, code and protocols have been provided within the article or through supplementary data files.

## Abbreviations

*6 mA*: N6-methyladenine
AMR: Antimicrobial resistance
CTC: Connectionist temporal classification
HMW: High-molecular weight
MADI-TOF: Matrix-assisted laser desorption/ionization
NGS: Next-generation sequencing
ONT: Oxford nanopore technologies
RNN: Recurrent neuronal network
WGS: Whole genome sequencing
