# Supplementary material for: High quality genome assemblies of Mycoplasma bovis using a taxon-specific Bonito basecaller for MinION and Flongle long-read nanopore sequencing
Source: BMC Bioinformatics. 2020 Nov 11;21:517. doi: 10.1186/s12859-020-03856-0 (PMC7661149; doi:10.1186/s12859-020-03856-0)
Supplement: Supplementary file 1 — Additional file 1: Figure S1. In-depth analyses of de novo consensus genomes between the Canu and Flye assemblers, with or without four rounds of Racon. While both Canu and Flye result in a similar number of mismatches (per 100 kbp), significantly more indels (per 10 kbp) are observed when implementing the Flye assembler in the bioinformatics workflow. Decreased Flye performance is suggested to lie within decreased insertion accuracies as compared to Canu. Implementation of four rounds of Racon allows increased Flye insertion Q-scores when custom-pg45 basecalling is used. However, Canu without four rounds of Racon still results in an overall better performance. [file 12859_2020_3856_MOESM1_ESM.docx]

**Supplementary Figure 1: In-depth analyses of *de novo* consensus genomes between the Canu and Flye assemblers, with or without four rounds of Racon.** While both Canu and Flye result in a similar number of mismatches (per 100 kbp), significantly more indels (per 10 kbp) are observed when implementing the Flye assembler in the bioinformatics workflow. Decreased Flye performance is suggested to lie within decreased insertion accuracies as compared to Canu. Implementation of four rounds of Racon allows increased Flye insertion Q-scores when custom-*pg45* basecalling is used. However, Canu without four rounds of Racon still results in an overall better performance.
