# Supplementary material for: High quality genome assemblies of Mycoplasma bovis using a taxon-specific Bonito basecaller for MinION and Flongle long-read nanopore sequencing
Source: BMC Bioinformatics. 2020 Nov 11;21:517. doi: 10.1186/s12859-020-03856-0 (PMC7661149; doi:10.1186/s12859-020-03856-0)
Supplement: Supplementary file 2 — Additional file 2: Table S3. Sequencing summary of single M. bovis sequencing runs (24h) on Flongle R9.4.1 flow cells and resulting coverages after qcat and NanoFilt filtering and trimming. [file 12859_2020_3856_MOESM2_ESM.docx]

**Table S3: Sequencing summary of single *M. bovis* sequencing runs (24h) on Flongle R9.4.1 flow cells and resulting coverages after qcat and NanoFilt filtering and trimming.**

|  | **Available nanopores at start (of 126)** |  |  |  | **Coverage (fold)** | | |
| --- | --- | --- | --- | --- | --- | --- | --- |
| **Strain (Flongle)** |  | **N_50_ (bps)** | **Total bases (Mbps)** | **Total Reads (Guppy)** | **Guppy (v.3.3.0)** | **Bonito custom-*pg45* (v0.1.3)**  **(*Flongle*)** | **Bonito custom-*pg45* (v0.1.3)**  **(*MinION subsampled*)** |
| *M. bovis* PG45 | 82 | 6,662 | 132.4 | 37,602 | 132 | 97 | 105 |
| *M. bovis* Mb267 | 52 | 6,382 | 57.5 | 19,093 | 57 | 45 | 48 |
| *M. bovis* Mb240 | 26 | 5,783 | 60.3 | 19,003 | 60 | 43 | 41 |
| *M. bovis* Mb194 | 43 | 6,357 | 97.2 | 31,128 | 97 | 73 | 71 |
| *M. bovis* Mb183 | 70 | 6,093 | 154.2 | 44,166 | 154 | 103 | 103 |
| *M. bovis* Mb182 | 68 | 7,524 | 321.6 | 85,841 | 321 | 256 | 246 |
| *M. bovis* Mb168 | 69 | 6,818 | 95.5 | 26,772 | 95 | 73 | 85 |
| *M. bovis* Mb166 | 74 | 7,130 | 217.9 | 63,231 | 217 | 165 | 157 |
| *M. bovis* Mb152 | 62 | 6,272 | 118.2 | 36,214 | 118 | 95 | 93 |
| *M. bovis* Mb1 | 41 | 8,241 | 176.2 | 45,532 | 176 | 143 | 135 |
| Mean | 59 | 6,726 | 143.1 | 40,858 | 143 | 109 | 108 |
