# Supplementary material for: High quality genome assemblies of Mycoplasma bovis using a taxon-specific Bonito basecaller for MinION and Flongle long-read nanopore sequencing
Source: BMC Bioinformatics. 2020 Nov 11;21:517. doi: 10.1186/s12859-020-03856-0 (PMC7661149; doi:10.1186/s12859-020-03856-0)
Supplement: Supplementary file 3 — Additional file 3: Table S1. Sequencing summary of multiplexed M. bovis and E. coli sequencing runs (48 h) on MinION R9.4.1 flow cells and resulting coverages after qcat and NanoFilt filtering and trimming.. [file 12859_2020_3856_MOESM3_ESM.docx]

**Table S1: Sequencing summary of multiplexed *M. bovis* and *E. coli* sequencing runs (48h) on MinION R9.4.1 flow cells and resulting coverages after qcat and NanoFilt filtering and trimming.**

|  |  |  |  | **Coverage (fold)** | | |  |
| --- | --- | --- | --- | --- | --- | --- | --- |
| **Strain (MinION)** | **N_50_ (bps)** | **Total bases (Mbps)** | **Total Reads (Guppy)** | **Guppy (v.3.3.0)** | **Bonito custom-*pg45* (v.0.1.3)** | **Bonito default dna_r9.4.1 (v.0.1.3)*** |  |
| *E. coli* ATCC25922 | 5,858 | 1,650.1 | 535,607 | 317 | 288 | 306 |  |
| *M. bovis* PG45 | 4,709 | 309.7 | 147,598 | 309 | 268 | 284 |  |
| *M. bovis* Mb267 | 5,563 | 431.9 | 153,314 | 430 | 356 | *NA* |  |
| *M. bovis* Mb240 | 3,382 | 188.3 | 114,477 | 188 | 165 | *NA* |  |
| *M. bovis* Mb194 | 5,271 | 399.1 | 173,514 | 398 | 352 | *NA* |  |
| *M. bovis* Mb183 | 4,177 | 500.1 | 227,003 | 498 | 425 | *NA* |  |
| *M. bovis* Mb182 | 6,181 | 281.3 | 101,82 | 280 | 246 | *NA* |  |
| *M. bovis* Mb168 | 7,076 | 604.8 | 191,199 | 603 | 512 | *NA* |  |
| *M. bovis* Mb166 | 5,350 | 230.3 | 92,311 | 229 | 195 | *NA* |  |
| *M. bovis* Mb152 | 7,251 | 926.8 | 302,933 | 924 | 840 | *NA* |  |
| *M. bovis* Mb1 | 9,476 | 955.8 | 184,195 | 953 | 851 | *NA* |  |
| Mean *M. bovis* | 5,843 | 482.8 | 168,836 | 481 | 421 | *NA* |  |
| **Bonito dna_r9.4.1 model was only used in comparative analysis with E. coli ATCC 25922.*  *NA; Not Applicable* | | | | | | | |
|  | | | | | | | |
|  | | | | | | | |
