# Supplementary material for: High quality genome assemblies of Mycoplasma bovis using a taxon-specific Bonito basecaller for MinION and Flongle long-read nanopore sequencing
Source: BMC Bioinformatics. 2020 Nov 11;21:517. doi: 10.1186/s12859-020-03856-0 (PMC7661149; doi:10.1186/s12859-020-03856-0)
Supplement: Supplementary file 4 — Additional file 4: Table S2. Sequencing summary of the M. bovis MiSeq (Illumina) sequencing run with resulting genome coverages after quality-filtering and adapter-clipping. [file 12859_2020_3856_MOESM4_ESM.docx]

**Table S2: Sequencing summary of the *M. bovis* MiSeq (Illumina) sequencing run with resulting genome coverages after quality-filtering and adapter-clipping.**

| **Strain (MiSeq)*** | **Total Reads** | **Total bases (Mbps)** | **Coverage (fold)** |  |  |  | |
| --- | --- | --- | --- | --- | --- | --- | --- |
| *M. bovis* PG45 | 17,741,780 | 2,679.0 | 2670 |  |  |  | |
| *M. bovis* Mb267 | 17,684,484 | 2,670.4 | 2661 |  |  |  | |
| *M. bovis* Mb240 | 18,640,154 | 2,814.7 | 2805 |  |  |  | |
| *M. bovis* Mb194 | 17,042,934 | 2,573.5 | 2565 |  |  |  | |
| *M. bovis* Mb183 | 18,450,972 | 2,786.1 | 2777 |  |  |  | |
| *M. bovis* Mb182 | 15,712,646 | 2,372.6 | 2365 |  |  |  | |
| *M. bovis* Mb168 | 22,287,562 | 3,365.4 | 3354 |  |  |  | |
| *M. bovis* Mb166 | 15,540,842 | 2,346.7 | 2339 |  |  |  | |
| *M. bovis* Mb152 | 16,899,142 | 2,551.8 | 2543 |  |  |  | |
| *M. bovis* Mb1 | 17,946,820 | 2,710.0 | 2701 |  |  |  | |
| Mean | 17,794,734 | 2,687.0 | 2678 |  |  |  | |
| **Paired-end reads were combined* | | | | | | |  |
